# *Acanthopanax henryi*: Review of Botany, Phytochemistry and Pharmacology

**DOI:** 10.3390/molecules26082215

**Published:** 2021-04-12

**Authors:** Xiao-Jun Li, Si-Qi Tang, Hao Huang, Jiao Luo, Xiao-Dan Zhang, Chang-Soo Yook, Wan-Kyunn Whang, Youn-Chul Kim, Xiang-Qian Liu

**Affiliations:** 1 National Engineering Research Center for Modernization of Traditional Chinese Medicine-Hakka Medical Resources Branch, School of Pharmacy, Gannan Medical University, Ganzhou 341000, China; xjli@gmu.edu.cn (X.-J.L.); sugarysiqi@163.com (S.-Q.T.); huanghao_26@163.com (H.H.); 2 School of Pharmacy, Hunan University of Chinese Medicine, Changsha 410208, China; luojiaojiao@stu.hnucm.edu.cn; 3 College of Life Sciences, Zhejiang Sci-Tech University, Hangzhou 310018, China; zxd_211@aliyun.com; 4 School of Pharmacy, KyungHee University, Seoul 130-701, Korea; yookcs@khu.ac.kr; 5 Department of Global Innovative Drugs, The Graduate School of Chung-Ang University, Seoul 156-756, Korea; whang-wk@cau.ac.kr; 6 College of Pharmacy, Wonkwang University, Iksan 54538, Korea

**Keywords:** *Acanthopanax henryi*, *Eleutherococcus henryi*, botany, phytochemistry, pharmacology, biological activities

## Abstract

*Acanthopanax henryi* (Oliv.) Harms (Araliaceae), also known as *Eleutherococcus henryi* and *Caoyewujia* (*Hengliwujia*) in Chinese, is a widely used traditional Chinese herb with the effects of expelling wind and removing dampness, relaxing the muscles and stimulating the blood circulation, and regulating the flow of *qi* to alleviate pain in the theory of Traditional Chinese Medicine. *Acanthopanax henryi* (AH, thereafter) possesses ginseng-like activities and is known as ginseng-like herb. In the past decade, a great number of phytochemical and pharmacological studies on AH have been carried out. Several kinds of chemical compositions have been reported, including terpenoids (monoterpenoids, diterpenoids, and triterpenoid saponins), phenylpropanoids, caffeoyl quinic acid derivatives, flavonoids, lignans, sterols, fatty acids, etc., among which, triterpenoid saponins were considered to be the most active components. Considerable pharmacological experiments in vitro have demonstrated that AH possessed anti-neuroinflammatory, anti-adipogenic, anti-inflammatory, antibacterial, anti-cancer, anti-oxidation, anti-AChE, anti-BuChE, and antihyaluronidase activities. The present review is an up-to-date and comprehensive analysis of the botany, phytochemistry, and pharmacology of AH.

## 1. Introduction

*Acanthopanax* spp. plays an important role in traditional oriental medicine in China, Korea, Japan, and far-east Russia. Its dried roots and stem barks are famous traditional folk medicine for treating rheumatism, arthritis, paralysis, sinew, and bone pain [1]. *Acanthopanax* Miq. as an endemic Asian genus with over 100 species (including 54 species and 49 varieties) are mainly distributed in North-eastern Asia including China, Korea, and Japan [2]. In other regions such as Blutan, India, Mongol, Malaysia, Nepal, Philippines, Russia, Thailand, and Vietnam, *Acanthopanax* spp. are also found [3]. These medicinal plants belonging to *Acanthopanax* spp. (Araliaceae) possess ginseng-like activities and are known as ginseng-like herb [4], and they are widely used as traditional oriental medicine having tonic, anti-rheumatic, longitudinal bone growth, adaptogenic activity, anti-fatigue, anti-stress, and anti-ischemic heart disease benefits [5,6]. The root barks of the plants in *Acanthopanax* spp. have been listed in the official Pharmacopeia. The Chinese Pharmacopeia, revised in 2020, listed *Acanthopanacis Cortex* originated as dry root bark of *Acanthopanax gracilistylus* W.W. Smith. The Korean Pharmacopeia (10th edition) listed *Acanthopanics Cortex* as having originated as the root and stem bark of *Acanthopanax sessiliflorusm* Seeman and some other plants in the same species. The Japanese Pharmacopeia (17th edition), revised in 2016, listed *Eleutherococci senticosi* Rhizoma as having originated as the rhizome and root of *Eleutherococcus senticosus* (Ruprecht et Maximowicz) Maximowicz.

The crude extract of most medical plants from *Acanthopanax* spp. are reported as having various pharmacological effects such as anti-fatigue, immune-enhancing effect, anti-diabetic, and antidepressant effect, etc. The most studied species is *Acanthopanax senticosus*, which mainly offers benefits for anti-oxidative stress, anti-diabetic, anti-inflammatory, anti-oxidant, cardiac protection, and increase of bone turnover and bone mineral density, etc. Up to now, there are many reports concerning the chemical components (mainly lignans, terpenoids, phytosteroids, flavonoids, phenolic compounds, coumarins, and fatty acids) and their bioactivities of *Acanthopanax* spp. [7].

**Figure 1:**
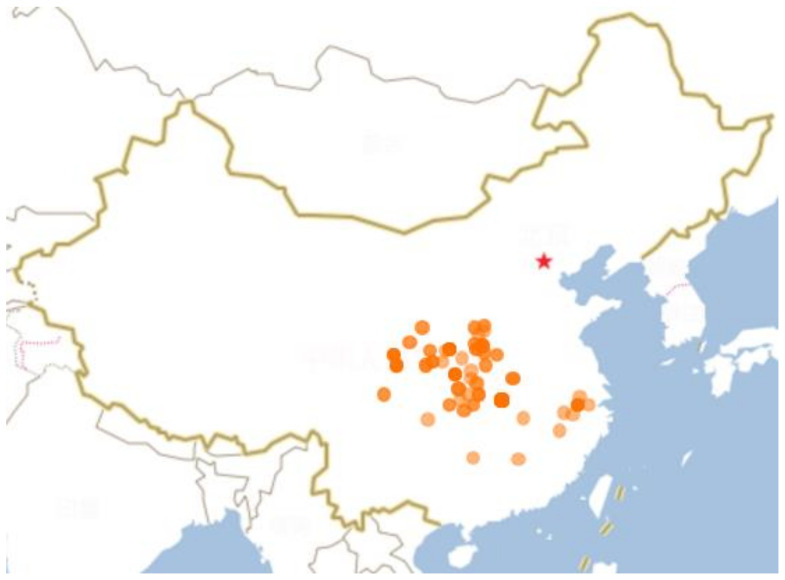
The producing area of *A. henryi* in China (Circles). The picture comes from Flora of China (http://www.eflora.cn; accessed on 07 December 2020). Star: Beijing.

*Acanthopanax henryi* (Oliv.) Harms belongs to Araliaceae which is widely distributed in the Provinces of Jiangxi, Hunan, Sichuan, Anhui, and Zhejiang in China (Figure 1). As a Chinese endemic plant, its dry root barks is listed officially in the provincial standard for traditional Chinese medicinal materials in Hunan (2009 edition) as *Acanthopanacis Cortex* (named as Wujiapi), which has been used as medicine for the treatment of paralysis, arthritis, rheumatism, lameness, and liver disease [1,8]. Previous phytochemical investigations of this plant have led to the isolation and identification of more than a hundred secondary metabolites, including monoterpenoids and their glycosides, diterpenoids, triterpenoid saponins, phenylpropanoids, caffeoyl quinic acid derivatives, flavonoids and their glycosides, lignans and their glycosides, steroids and their glycosides, and fatty acids which showed diverse biological activities, such as anti-neuroinflammatory, anti-obesity, anti-inflammatory, anti-tumor, anti-oxidation, anti-AChE, and antimicrobial effects. This review aims to provide an overview of botany, phytochemical components, and the pharmacological functions present in AH.

## 2. Botany

**Figure 2:**
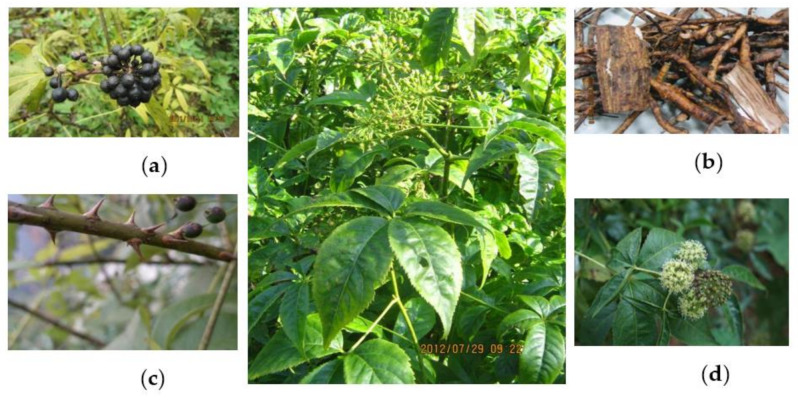
*Acanthopanax henryi* (Oliv.) Harms (AH). (Photographed by Prof. X.Q. Liu, in Loudi, Hunan Province of China in 2011 and 2012). (**a**): Fruits of AH. (**b**): Roots of AH. (**c**): Stems of AH. (**d**): Flowers of AH.

*Acanthopanax henryi* (Oliv.) Harms. (AH) is a hermaphroditic deciduous shrub or tree to 1–3 m tall (Figure 2), with sparse branches and anfractuous rough thorns; branchlets: densely covered with soft, short hair which then gradually falls off; leaves: palmately compound five foliate, rarely three, petioles 4–7 cm long, with bristled hair on both surfaces along veins; leaves like paper, close together on the short branch, are in leaflets of five, elliptic or narrowly oblong, rarely obovate, apex acute or acuminate, base narrow wedge-shaped, 8–12 cm long, 3–5 cm wide, dark green above, rough, gray-green below, leaf veins covered with soft, short hair, leaf margins and above middle part with small serrate, lateral veins in 6–8 pairs, both sides of leaves are raised and obvious but the leaf veins are not obvious; small petioles are 3–6 mm long, with thick, short hair, sometimes with no small petiole; flowers: smaller, umbel inflorescence, 1.5–2.5 cm in diameter, the total pedicel robust and with thick, short hair which then gradually falls off, five petals, five stamens, five ovaries at the base, the style all together generate columnar, blooming from July to September; fruits: drupe, globular, black-ripen from September to October; seeds: two in each fruit, small and flat. AH is grown widely in the provinces of Jiangxi, Shanxi, Shaanxi, Sichuan, Hubei, Henan, Anhui, and Zhejiang. It grows in the forest edge or bushes, at an altitude of 1000–3200 m [1].

AH has the effects of dispelling wind and dampness, promoting blood circulation and relaxing tendons, regulating *qi*, and relieving pain in the theory of Traditional Chinese Medicine. In folk medicine, it is mainly used in the treatment of rheumatic arthralgia, contracture and numbness, weakness of muscles and bones, edema, traumatic injury, rheumatoid arthritis, and hernia abdominal pain as well as related conditions [9,10].

## 3. Chemical Constituents

To date, the chemical components (Table 1) of AH mainly contain monoterpenoids and their glycosides, diterpenoids, triterpenoid saponins, phenylpropanoids and their glycosides, caffeoyl quinic acids, flavonoids, lignans and their glycosides, steroids, fatty acids, and other compounds. Of these compounds, monoterpenoids and their glycosides, triterpenoid saponins, caffeoyl quinic acids, and lignans and their glycosides as the major active components.

### 3.1. Monoterpenoids

**Figure 3:**
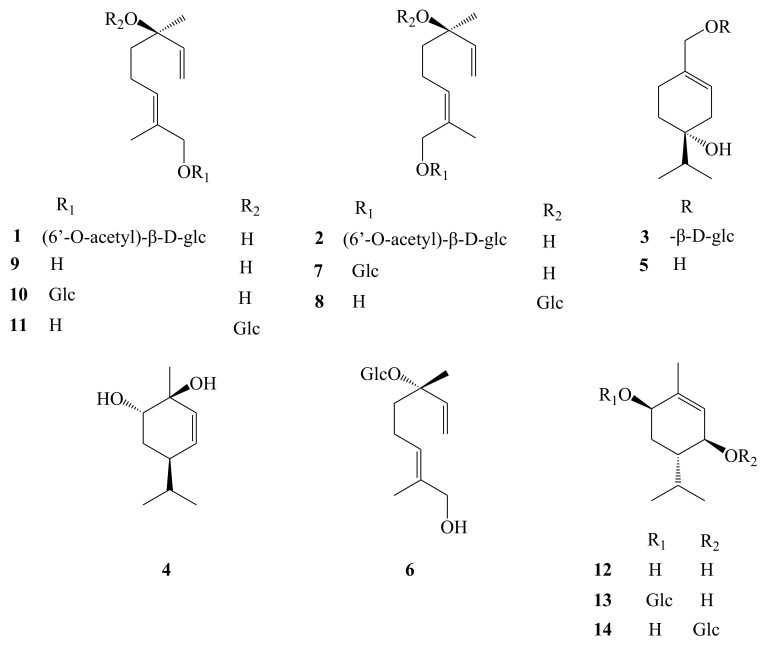
Chemical structures of monoterpenoids (**1**–**14**) identified from *A. henryi*.

AH contains abundant secondary metabolites; among them, the monoterpenes are the major ones, as listed in Table 1. To date, 14 of these compounds have been isolated from fruits of AH [11,12,13]. Of them, four are monoterpene aglycones, and ten are monoterpenoid glycosides (Figure 3). The above mentioned compounds include the three new monoterpenoid glycosides, (2*E*,6*R*)-6-hydroxy-2,6-dimethyl-2,7-octadien-1-yl-(6′-*O*-acetyl)-*O*-β-d-glucopyranoside (eleuhenryiside A, **1**), (2*Z*,6*R*)-6-hydroxy-2,6- dimethyl-2,7-octadien-1-yl-(6′-*O*-acetyl)-*O*-β-d-glucopyranoside (eleuhenryiside B, **2**), and (−)-(4*R*)-4,7-dihydroxy-1-menthene 7-O-β-d-glucopyranoside (eleuhenryiside C, **3**), which were isolated from the methanol extract of fruits of AH, based on our previous work [11]. The basic skeleton of these compounds can be divided into two types: linalool and menthene. The information of the monoterpenoids is shown in Table 1.

### 3.2. Diterpenoids

**Figure 4:**
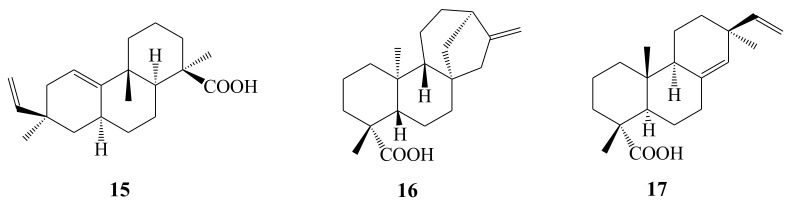
Chemical structures of diterpenoids (**15**–**17**) identified from *A. henryi*.

Compared with other kinds of compounds, three diterpene compounds from this plant were reported. Three diterpenoids, including acanthoic acid, kaurenoic acid, and pimaric acid, were isolated and purified from the roots of AH [14,15]. Moreover, the skeletons of these compounds are tricyclic (acanthoic acid, **15** and pimaric acid, **17**) and tetracyclic (kaurenoic acid, **16**) diterpenes (Figure 4).

### 3.3. Triterpenoid Saponins

**Figure 5:**
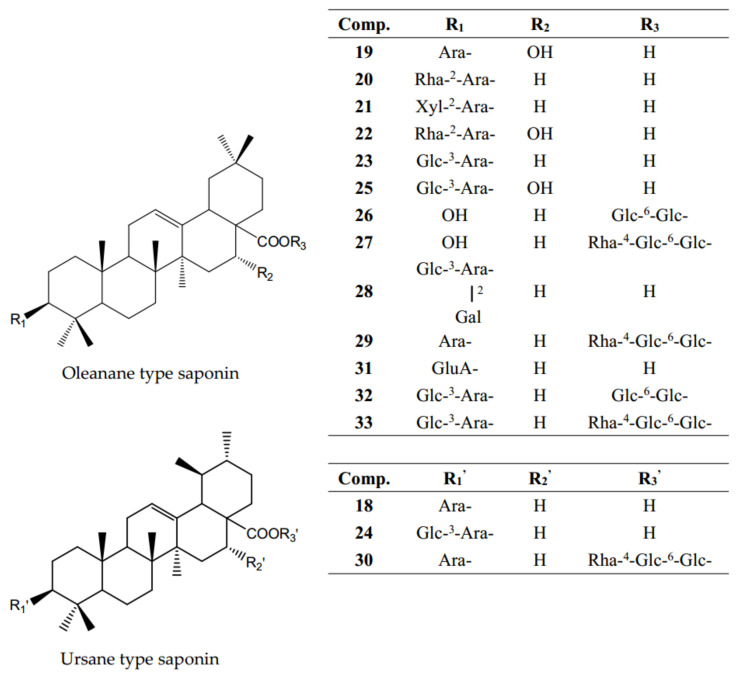
Chemical structures of triterpenoid saponins (**18**–**33**) identified from *A. henryi*.

Triterpenoid saponin is another large category of active components in AH. Up to now, 16 triterpenoid saponins have been discovered from AH, such as ursolic acid 3-*O*-α-*L*-arabinopyranoside (**18**), echinocystic acid 3-*O*-α-*L*-arabinopyranoside (**19**), eleutheroside K (**20**), prosapogenin CP_2b_ (**21**), tauroside D (**22**), guaianin N (Glycoside St-C1, **23**), matesaponin J_2_ (**24**), echinocystic acid 3-*O*-β-d-glucopyranosyl-(1→3)-*O*-α-*L*-arabinopyranoside (**25**), hemslonin A (**26**), cussonoside B (**27**), oleanolic acid 3-*O*-[β-d-glucopyranosyl-(1→3)]-β-d- galactopyranosyl-(1→2)-*O*-α-*L*-arabinopyranoside (Glycoside St-E2, **28**), ciwujianoside C3 (**29**), ursolic acid 3-*O*-α-*L*-arabinopyranosyl-28-*O*-α-L-rhamnopyranosyl-(1→4)-*O*- β-d-glucopyranosyl-(1→6)-*O*-β-d-glucopyranoside (**30**), oleanolic acid-3-*O*-β-d- glucuronopyranoside (**31**), araliasaponin II (**32**), and begoniifolide A (**33**) [16,17,18] (Figure 5). The basic skeleton of these saponins can be divided into two types: oleanane and ursane. Among the 16 compounds, two major triterpenoid ingredients are guaianin N (**23**) and matesaponin J_2_ (**24**), which are a pair of isomers [16,17,18]. In addition, among these triterpenoid saponins, the sugar groups are generally located at C-3 or/and C-28. The information on the triterpenes is shown in Table 1.

### 3.4. Phenylpropanoids

**Figure 6:**
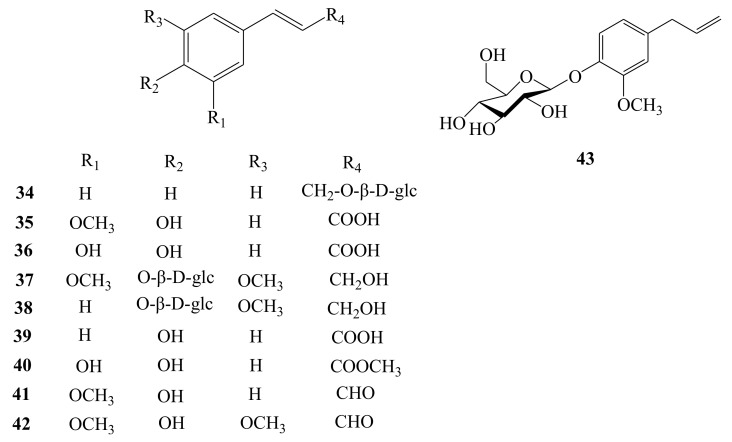
Chemical structures of phenylpropanoids (**34**–**43**) identified from *A. henryi*.

Up to now, there are ten phenylpropanoids and their glycosides discovered from this plant. Rosin (**34**) and eugenol glucoside (**43**) were isolated from fruits and flowers of AH, respectively [12,21]. Ferulic acid (**35**), syringin (**37**), and *trans*-coniferin (**38**) have been obtained from the roots of AH [19]. Meanwhile, *trans*-*p*-hydroxycinnamic acid (**39**), (*E*)-caffeic acid methyl ester (**40**), *trans*-coniferyl aldehyde (**41**), and *trans*-sinapaldehyde (**42**) were retrieved from the stem part of this plant [20]. Additionally, caffeic acid (**36**) was purified from both roots and stems of AH [19,20] (Figure 6).

### 3.5. Caffeoyl Quinic Acids

**Figure 7:**
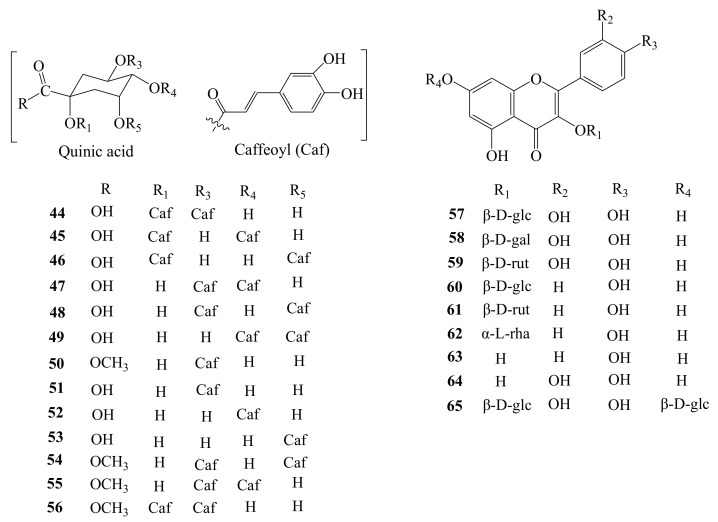
Chemical structures of caffeoyl quinic acids (**44**–**56**) and flavonoids (**57**–**65**) identified from *A. henryi*.

AH also contains rich caffeoyl quinic acid derivatives compared with the medicinal plants which belong to the same genus, *Acanthopanax* Miq, such as *A. gracilistylus*, *A. senticosus*, and *A. sessiliflorus*. Using the method of column chromatography, 13 caffeoyl quinic acids have been separated and purified from AH. It has been shown that four of them are caffeoyl monosubstituted compounds (**50**–**53**), and the other nine are caffeoyl disubstitutes and their methyl esters (**44**–**49**, **54**–**56**) (Figure 7). This type of compound exists throughout the plant, including fruit [13], root [19], stem [20], flower [21], and leaf [22]. The information on the caffeoyl quinic acids is shown in Table 1.

### 3.6. Flavonoids

In the previous report, nine flavonoids and their glycosides were isolated from the fruit, flower, and leaf of AH. They are quercetin-3-*O*-β-d-glucopyranoside (**57**), quercetin-3-*O*-β-d-galactopyranoside (**58**), rutin (**59**), kaempferol-3-*O*-β-d-glucoside (**60**), kaempferol-3-rutinoside (**61**), kaempferol-3-*O*-α-*L*-rhamnoside (**62**), kaempferol (**63**), quercetin (**64**), and quercetin-3,7-di-β-*O*-glucopyranoside (**65**) [12,13,21,22,23]. Their molecular skeletons are divided into two types: quercetin and kaempferol. Generally, one or two sugars are connected (Figure 7).

### 3.7. Lignans

**Figure 8:**
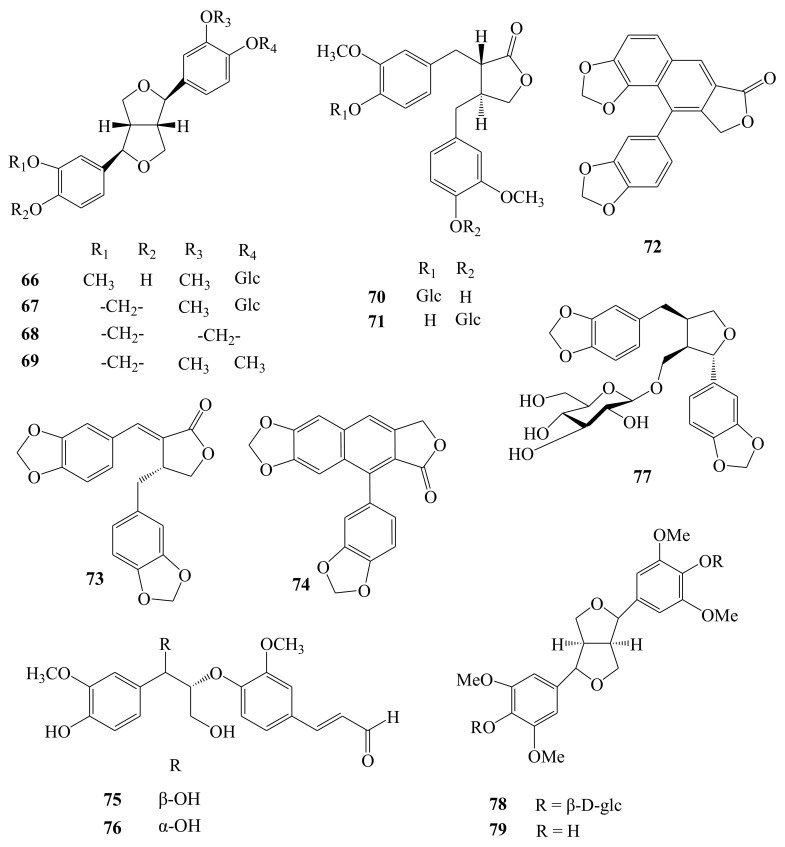
Chemical structures of lignans (**66**–**79**) identified from *A. henryi*.

Lignans have been suggested to be evolutionarily derived by elaboration of the phenylpropanoid pathway for a plant’s own benefits, as its immunoprotection and protection, as it were, from harmful free radicals [27]. According to our previous work, 14 lignans and their glycosides were isolated and purified from the fruit [11,12], root [15,19,24,25], stem [20], and flower [21] of this plant, including (−)-pinoresinol 4-*O*-β-d-glucopyranoside (**66**), (+)-simplexoside (**67**), (−)-sesamin (**68**), (−)-kobusin (**69**), styraxlignolide E (**70**), styraxlignolide D (**71**), helioxanthin (**72**), savinin (**73**), taiwanin C (**74**), (+)-*threo*-(7*R*,8*R*)-guaiacylglycerol-β-coniferyl aldehyde ether (**75**), (+)-*erythro*-(7*S*,8*R*)-guaiacylglycerol-β-coniferyl aldehyde ether (**76**), dihydrosesamin-9-*O*-β-d-glucopyranoside (**77**), syringaresinol diglucoside (Eleutheroside E, **78**), and syringaresinol (**79**) (Figure 8). Of them, (−)-sesamin is one of the main active ingredients [12,19,20]. Several studies revealed that (−)-sesamin possesses potent anti-cancer properties. The anti-cancer effects of sesamin have been mainly attributed to its anti-proliferative, pro-apoptotic, anti-inflammatory, anti-metastatic, anti- and pro-angiogenic, and pro-autophagocytic activities. Also, the previous researches indicate that NF-κB, STAT3, JNK, ERK1/2, p38 MAPK, PI3K/AKT, caspase-3, and p53 signaling pathways are critically involved in mediating the anti-cancer effects of (−)-sesamin [28]. In addition, oral sesamin administration (50 mg/kg·bodyweight/day) significantly attenuated depressive, aversive, repetitive, and anxiety-like behaviors in a long-term multiple nonsocial stress-treated CD-1 mice model. Sesamin inhibited stress-induced gut barrier integrity damage, reduced circulating lipopolysaccharide (LPS) levels, and suppressed neuroinflammatory responses. Moreover, sesamin treatment also restructured the gut microbiome by enhancing the relative abundances of Bacteroidales and S24-7. The correlation analysis indicated that the microbiota composition changes were strongly correlated with behavioral disorders, serotonin, norepinephrine, and LPS levels. In conclusion, sesamin has preventive effects on stress-induced behavioral and psychological disorders, which might be highly related to the reshaped microbiota composition [29].

### 3.8. Steroids

**Figure 9:**
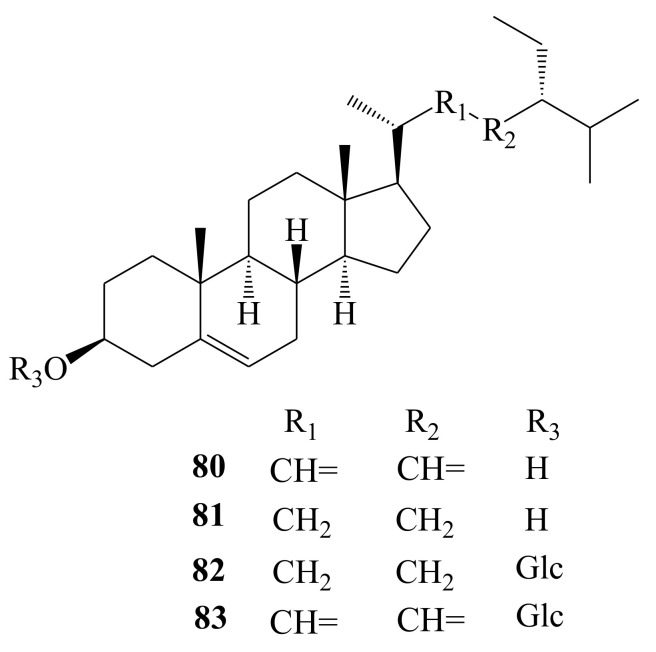
Chemical structures of steroids (**80**–**83**) identified from *A. henryi*.

The major steroidal compounds in AH are stigmasterol (**80**) [15,19,20,23], β-sitosterol (**81**) [15,19,20,24], daucosterol (**82**) [14,23], and stigmasterol-3-O-β-d-glucopyranoside (**83**) [26] (Figure 9). Their detailed information is shown in Table 1.

### 3.9. Fatty Acids

**Figure 10:**
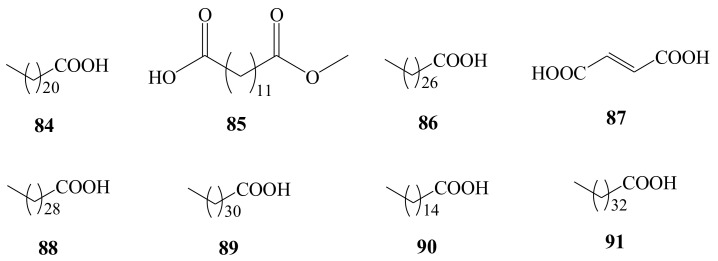
Chemical structures of fatty acids (**84**–**91**) identified from *A. henryi*.

Using the methods of column chromatography for the separation and purification of AH, eight organic acids were obtained. Of them, seven are long-chain fatty acids, including behenic acid (**84**) [19], undecanedioic acid, monomethyl ester (**85**) [20], octacosanic acid (**86**) [24], melissic acid (**88**), lacceroic acid (**89**), palmitic acid (**90**), and gheddic acid (**91**) [23]. One is unsaturated diacid (fumaric acid, **87**) [16] (Figure 10).

### 3.10. Other Compounds

**Figure 11:**
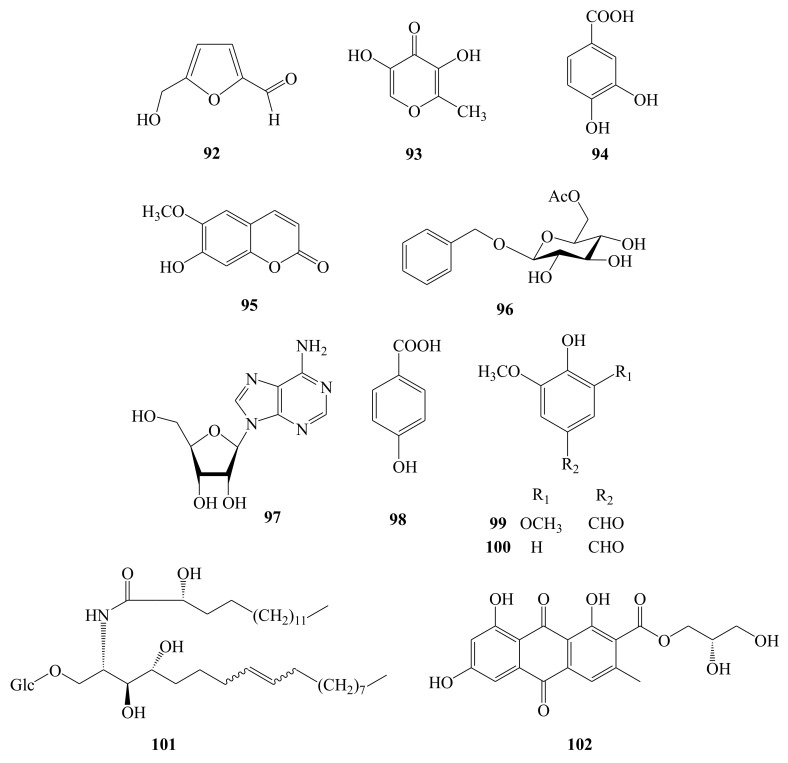
Chemical structures of other compounds (**92**–**102**) identified from *A. henryi*.

5-hydroxymethyl-2-furaldehyde (**92**), 5-hydroxymaltol (**93**), protocatechuic acid (**94**), 6-methoxy-7-hydroxycoumarin (**95**) [12,19,20], phenylmethyl-β-d-glucopyranoside-6′-*O*- acetate (**96**) [12], adenosine (**97**) [19], *p*-hydroxybenzoic acid (**98**), syringaldehyde (**99**), vanillin (**100**) [20], 1-O-β-d-glucopyranosyl-(2*S*,3*S*,4*R*,8*E*/*Z*)-2- (2′-hydrooxypalmitoylamino)-8-octadecene-1,3,4-triol (**101**), and glyceroyl-1,6,8- trihydroxy-3-methyl-9,10-dioxo-2-anthracene carboxylate (**102**) [16] were also isolated from different medicinal parts of AH (Figure 11). More detailed information is listed in Table 1.

## 4. Pharmacology Research

The traditional functions of AH are for paralysis, arthritis, rheumatism, lameness, edema, injuries from fall, hernia, and abdominal pain applications. According to its traditional efficacies, researchers have conducted a series of studies and found that the plant also possesses anti-neuroinflammatory, anti-adipogenic, anti-inflammatory, antibacterial, anti-cancer, anti-oxidation, anti-AChE, anti-BuChE, and antihyaluronidase functions (Table 2). The active ingredients related to these functions are mainly lignans, pentacyclic triterpenoid saponins, flavonoids, and caffeoyl quinic acid derivatives.

### 4.1. Anti-Neuroinflammatory Activity

In our previous study, the lignan savinin (**73**), with anti-neuroinflammatory activity, was isolated from the roots of AH. Savinin showed inhibitory activity against lipopolysaccharide (LPS)-induced nitric oxide (NO) and prostaglandin E_2_ (PGE_2_) production with IC_50_ values of 2.22 ± 0.11 and 2.28 ± 0.23 μM, respectively. The effects of savinin were associated with the suppression of LPS-induced expression of the inducible nitric oxide synthase (iNOS) and cyclooxygenase-2 (COX-2) protein. Furthermore, savinin negatively regulated the production of interleukin (IL)-1β and tumor-necrosis factor (TNF)-α at the transcriptional level in LPS-stimulated BV2 microglial cells. These anti-neuroinflammatory effects of savinin were mediated by p38 mitogen-activated protein kinase (MAPK), but not ERK or JNK MAPKs [19].

In addition, the phytochemical investigation on the fruits of AH resulted in the discovery of three novel monoterpene glycosides: eleuhenryiside A (**1**), eleuhenryiside B (**2**), and eleuhenryiside C (**3**), as well as a known lignan, (−)-kobusin (**69**). The anti-neuroinflammatory activities of these compounds were evaluated with LPS-stimulated BV2 microglia. The results showed that new compounds, eleuhenryiside A and eleuhenryiside C, have inhibitory effects on NO production with IC_50_ values of 32.50 ± 1.60 and 3.54 ± 0.20 μM in LPS-stimulated BV2 microglia. Also, the lignan (−)-kobusin has ability to inhibit NO production with the IC_50_ values of 14.25 ± 2.69 μM in BV2 cells [11].

Also, utilizing LPS-induced microglia BV2 as the bioactivity-guided model, 18 compounds were purified from ethyl acetate extract of methanol extract of AH fruits. The results of screening of anti-neuroinflammatory activity demonstrated that the tested compounds showed certain NO inhibitory effects. Among them, 5-hydroxymethyl-2-furaldehyde (**92**), 6-methoxy-7-hydroxycoumarin (**95**), (−)-pinoresinol 4-*O*-β-d-glucopyranoside (**66**), (−)-sesamin (**68**), 3,4-dihydroxy-*p*-menth-1-ene (**4**), (4*R*)-*p*-menth-1-en-4,7-diol (**5**), and styraxlignolide D (**71**) showed moderate NO inhibitory effects, with the inhibition values at 41.5%, 46.8%, 42.9%, 42.2%, 40.7%, 49.3%, and 47.8% in 80 μM, respectively [12].

Our previous work showed that the methanol extracts of flowers and stems of AH as well as their ethyl acetate extraction part could inhibit the production of NO in LPS-stimulated BV2 microglia, respectively [20,21].

### 4.2. Anti-Adipogenic Effects

A study showed that the pentacyclic triterpenoid saponins Glycoside St-C1 (**23**) and Glycoside St-E2 (**28**), isolated from the leaves of AH, decreased lipid accumulation in 3T3-L1 cells, and these results were related to the inhibition of peroxisome proliferator-activated receptor γ (PPARγ) and CCAAT/enhancer-binding protein α (C/EBPα). Additionally, Glycoside St-C1 and Glycoside St-E2 induced phosphorylation of AMP-activated protein kinase (AMPK), which played a crucial role of an upstream factor of PPARγ and C/EBPα. In short, Glycoside St-C1 and Glycoside St-E2 can inhibit adipogenesis through the AMPK-PPARγ-C/EBPα mechanism [30].

### 4.3. Anti-Inflammatory Activity

Some studies have reported that triterpenoid saponins isolated from the leaves of AH had a significant anti-inflammatory effect [11,20,21,31,32,33]. For instance, Ciwujianoside C3 (**29**) exhibited no cytotoxicity at the measured concentrations in RAW264.7 macrophages. Treatment with Ciwujianoside C3 inhibited NO production, proinflammatory cytokine levels, including IL-6, TNF-α, and PGE_2_, and protein and mRNA expression levels of iNOS and COX-2. Furthermore, Ciwujianoside C3 suppressed phosphorylation of extracellular signal-regulated kinases and c-jun N-terminal kinases. It was also able to suppress activation of NF-κB via inhibition of the TLR4 signaling pathway. In brief, Ciwujianoside C3 exerts inhibitory effects on LPS-induced PGE_2_, NO, IL-6, and TNF-α production. In addition, iNOS and COX-2 expressions were decreased in RAW264.7 murine macrophages. These inhibitory effects may be achieved via suppression of MAPKs and NF-κB phosphorylation following inhibition of the TLR4 signaling pathway [31].

In the cell model mentioned above, Araliasaponin II (**32**) markedly inhibited the production of NO and PGE_2_ and reduced iNOS and COX-2 expression at the transcriptional and translational levels. Also, it downregulated the expression of interleukin-6 and tumor necrosis factor-α at the protein and mRNA levels. Furthermore, pre-treatment with Araliasaponin II significantly suppressed the TLR-4-NF-κB signaling pathway; this effect may be caused by Araliasaponin II competing with LPS for binding to TLR-4 and subsequently inhibiting translocation of the NF-κB/p65 protein to the nucleus [32].

In our previous study, the rare anthraquinone, glyceroyl-1,6,8-trihydroxy-3-methyl- 9,10-dioxo-2-anthracene carboxylate (**102**), which was isolated from leaves of AH, significantly decreased the production of NO and the levels of other inflammatory factors, such as TNF-α and IL-6, in LPS-stimulated RAW264.7 macrophages in a dose-dependent manner. This is the first report of the anti-inflammatory effect of this compound [16]. Additionally, the lignan (−)-kobusin (**69**) purified from the fruits of AH showed abilities to inhibit NO production and had no influence on cell viability, with the IC_50_ value of 36.35 ± 6.27 μM in RAW264.7 cells [11].

Recent research reported that the hot methanol extract of the root bark of AH was subjected to XAD-4 column chromatography eluting with a gradient of methanol in water. The cytotoxicity and anti-inflammatory effects of the MeOH fractions were evaluated on the inhibition on LPS-induced nitric oxide, prostaglandin E_2_, interleukin-1β, and interleukin-6 production in RAW264.7 macrophages. Results showed that the 80% MeOH fraction was a better inhibitor of LPS-induced NO, PGE_2_, IL-1β, and IL-6 production, and expression of iNOS at the protein levels in a concentration-dependent manner [33].

Additionally, our previous studies have shown that the MeOH extracts of flowers and stems of AH, as well as their petroleum ether, ethyl acetate, and *n*-butanol extraction parts, had a potential anti-inflammatory activity based on the inhibition of NO production in LPS-induced RAW264.7 cells, respectively [20,21].

### 4.4. Antimicrobial Activity

A recent study showed that the pentacyclic triterpenoid saponin, ursolic acid 3-*O*-α-*L*-arabinopyranoside (URS, **18**), isolated from the leaves of AH, has antibacterial activity in combination with oxacillin (OXA) against methicillin-resistant *Staphylococcus aureus* (MRSA). The synergistic effects of URS and OXA were determined using a checkerboard dilution test and time-kill curve assay. The minimal inhibitory concentration (MIC) value of URS against MRSA was found to be 6.25 µg/mL and there was a partial synergistic effect between OXA and URS. The time-kill growth curves were suppressed by OXA combined with URS at a sub-inhibitory level. Compared to the optical density at 600 nm (OD600) value of URS alone (0.09 µg/mL), the OD600 values of the suspension in the presence of 0.09 µg/mL URS and 0.00001% Triton X-100 or 250 µg/mL N,N′-dicyclohexylcarbodiimide reduced by 56.6 and 85.9%, respectively. The transmission electron microscopy (TEM) images of MRSA indicated damage to the cell wall, broken cell membranes, and cell lysis following treatment with URS and OXA. Moreover, an inhibitory effect on the expression of penicillin-binding protein 2a (PBP2a) protein was observed when cells were treated with URS and OXA compared with untreated controls [34].

Moreover, one latest study reported that another pentacyclic triterpenoid saponin, Eleutheroside K (**20**), isolated from the leaves of AH, can effectively inhibit the growth of MRSA through down-regulating the expression of PBP2a [35].

### 4.5. Anticancer/Antitumor Activities

A recent study showed that the 100% MeOH fraction (after MeOH extract of AH was chromatographed on XAD-4 column) of the root bark of AH has moderate inhibitory effects on HL-60 human leukemia cells, HT-29 human colon cancer cells, and A549 human lung cancer cells with IC_50_ values of 50.41, 102.05, and 147.57 μg/mL, respectively [14]. Besides this, the 75% MeOH extract of roots of *E. henryi* cultivated in Poland exhibited moderate inhibition effect of HL-60 cell line growth with an IC_50_ value of 270 μg/mL [36].

### 4.6. Anti-Oxidant, Anti-AChE, and Anti-BuChE Activities

In recent years, a study was focused on the chemical constituents of the leaves of AH along with their antioxidant and acetyl cholinesterase (AChE) inhibitory activities. Caffeoyl quinic acid derivates and flavonoids were obtained from AH through column chromatography technologies, and the content of major constituents was determined by the HPLC-UV method. Anti-oxidant activity of the isolated metabolites was analyzed by free radical scavenging (DPPH, ABTS radicals) and superoxide anion scavenging. The results showed that antioxidant activity of di-caffeoyl quinic acid derivates, including 1,5-di-*O*-caffeoyl quinic acid (**46**), 3,4-di-*O*-caffeoyl quinic acid (**47**), 3,5-di-*O*-caffeoyl quinic acid (**48**), and 4,5-di-*O*-caffeoyl quinic acid (**49**) was stronger than that of the positive controls (ascorbic acid, trolox, and allopurinol). AChE inhibitory activity of the constituents was estimated. Among which, 4,5-di-*O*-caffeoyl quinic acid (49), 4-*O*-caffeoyl quinic acid (52), and quercetin (64) were found to have strong AChE inhibitory activity with IC_50_ values ranging from 62.6 to 121.9 μM. This study showed that some of the tested components from the leaves of AH exhibit strong anti-oxidant and anti-AChE activities in vitro [22].

A study showed that the 75% methanol extract of roots of *A. henryi* cultivated in Poland exhibited certain anti-AChE and anti-DPPH activities, with inhibitory rates at 19.6% (at the concentration of 100 μg/0.19 mL of the reaction mixture) and 14.7% (in 90 min), respectively. The extract reduced DPPH in a time-dependent mode of 90 min, at the concentration of 0.8 mg/mL [36].

Another work reported that, using the HPTLC assay method, the 75% ethanol extract of *A. henryi* exhibited certain anti-AChE and anti-BuChE activities with IC_50_ values of 1.75 ± 0.1 and 0.13 ± 0.02 mg/mL, respectively. Also, the chloroform extract of *A. henryi* showed weak BuChE inhibitory activity with an IC_50_ value of 1.21 ± 0.2 mg/mL. Moreover, the 75% ethanol and chloroform extracts of *A. henryi* exhibited anti-DPPH antioxidant activities [37].

Załuski and Janeczko reported that the ethanol extracts from fruits of *A. henryi*, freshly dried and stored for one year, had the effects of anti-DPPH with the EC_50_ values of 0.2 ± 0.01 and 0.23 ± 0.02 mg/mL, respectively [38].

### 4.7. Anti-Hyaluronidase Activity

A recent study reported that the 75% methanol extract of roots of *A. henryi* cultivated in Poland exhibited moderate antihyaluronidase activity, and the inhibitory rate was 40.7% at the concentration of 100 μg/0.16 mL of the reaction mixture [36]. In addition, the ethanol extracts from the fruit of *A. henryi*, after being freshly dried and stored for one year, had the effects of antihyaluronidase with the IC_50_ values of 0.61 ± 0.05 and 0.70 ± 0.04 mg/mL, respectively [38].

## 5. Discussion

The research findings indicated that the main chemical constituents of AH are monoterpenoids and their glycosides, triterpenoid saponins, caffeoyl quinic acid, and lignans. As to *A. gracilistylus*, included in Chinese Pharmacopoeia (Edition 2020), its major chemical components are diterpenoids, triterpenoids and their saponins, and ceramides [39]; *A. senticosus*, another species of traditional medicinal plant of *Acanthopanax*, has been widely studied by domestic and foreign scholars. A large number of documents reported that the main chemical constituents of *A. senticosus* are triterpenoid saponins and lignans [27]. Comparison of the three species of *Acanthopanax* mentioned above found that although they have similarities in traditional usage, such as they can be used to treat rheumatic arthralgia, contracture and numbness, weakness of muscles and bones, asthenia, and edema, etc., there are some differences in their main chemical components. Whether they can replace each other needs to be confirmed by more in-depth pharmacological and clinical studies. Therefore, they should be distinguished from each other when they are used. So far, there is no report on the essential oil of AH; further studies on the volatile components and their pharmacological activities of AH need to be carried out.

## 6. Conclusions

In summary, this review presents 102 chemical components of AH, mainly including monoterpenoids and their glycosides, diterpenoids, triterpenoid saponins, phenylpropanoids, caffeoyl quinic acid derivatives, flavonoids and their glycosides, lignans and their glycosides, sterols, fatty acids, and other compounds isolated from 1993 to 2021. Of them, triterpenoid saponins exhibit very good anti-inflammatory, anti-adipogenic, and antibacterial activities. The lignans showed potential anti-neuroinflammatory activity, and the extracts and fractions enriched in caffeoyl quinic acid derivatives and flavonoids have antioxidation and anti-AChE activities. The methanol and ethanol extracts showed anticancer, anti-BuChE, and antihyaluronidase activities.

However, the studies on pharmacological activities of crude extracts and isolated compounds are mostly focused on the cell level in vitro. A deeper investigation of isolated metabolites in preclinical and clinical studies are necessary to define the active constituents of AH. Therefore, further evaluation of in vivo activities of isolated compounds is urgently needed. Meanwhile, the discovery of new metabolites is important and promising and can provide clues for the development of new drugs. In this paper, the current research status of AH was reviewed in order to provide some scientific theoretical basis and reference for the follow-up research of the Chinese endemic medicinal plant.

## Figures and Tables

**Table 1:** Chemical compounds isolated from Acanthopanax henryi.

Classification	NO.	Chemical Component	Chemical Formula	Part of Plant	Ref.
Monoterpenoids	1	Eleuhenryiside A (new)	C_18_H_30_O_8_	Fruit	[11]
	2	Eleuhenryiside B (new)	C_18_H_30_O_8_	Fruit	[11]
	3	Eleuhenryiside C (new)	C_16_H_28_O_7_	Fruit	[11]
	4	3,4-dihydroxy-*p*-menth-1-ene	C_10_H_18_O_2_	Fruit	[12]
	5	(4*R*)-*p*-Menth-1-en-4,7-diol	C_10_H_18_O_2_	Fruit	[12]
	6	(2*E*,6*S*)-1-hydroxy-2,6-dimethyl-2,7-octadien-6-yl-β-d-glucopyranoside	C_16_H_28_O_7_	Fruit	[13]
	7	(2*Z*,6*R*)-6-hydroxy-2,6-dimethyl-2,7-octadien-1-yl-β-d-glucopyranoside	C_16_H_28_O_7_	Fruit	[13]
	8	(2*Z*,6*R*)-1-hydroxy-2,6-dimethyl-2,7-octadien-6-yl-β-d-glucopyranoside	C_16_H_28_O_7_	Fruit	[13]
	9	(2*E*,6*R*)-2,6-dimethyl-2,7-octadiene-1,6-diol	C_10_H_18_O_2_	Fruit	[12]
	10	(2*E*,6*R*)-6-hydroxy-2,6-dimethyl-2,7-octadien-1-yl-β-d-glucopyranoside	C_16_H_28_O_7_	Fruit	[13]
	11	(2*E*,6*R*)-1-hydroxy-2,6-dimethyl-2,7-octadien-6-yl-β-d-glucopyranoside	C_16_H_28_O_7_	Fruit	[13]
	12	(+)-(3*S*,4*S*,6*R*)-3,6-dihydroxy-1-menthene	C_10_H_18_O_2_	Fruit	[12]
	13	(−)-(3*S*,4*S*,6*R*)-3,6-dihydroxy-1-menthene 6-*O*-β-d-glucopyranoside	C_16_H_28_O_7_	Fruit	[13]
	14	(−)-(3*S*,4*S*,6*R*)-3,6-dihydroxy-1-menthene 3-*O*-β-d-glucopyranoside	C_16_H_28_O_7_	Fruit	[13]
Diterpenoids	15	Acanthoic acid	C_20_H_30_O_2_	Root	[14]
	16	Kaurenoic acid	C_20_H_30_O_2_	Root	[14]
	17	Pimaric acid	C_20_H_30_O_2_	Root	[15]
Triterpenoid saponins	18	Ursolic acid 3-*O*-α-*L*-arabinopyranoside	C_35_H_56_O_7_	Leaf	[16,17,18]
	19	Echinocystic acid 3-*O*-α-*L*-arabinopyranoside	C_35_H_56_O_8_	Leaf	[16,17,18]
	20	Eleutheroside K	C_41_H_66_O_11_	Leaf	[16,17,18]
	21	Prosapogenin CP_2b_	C_40_H_64_O_11_	Leaf	[16,17,18]
	22	Tauroside D	C_41_H_66_O_12_	Leaf	[16,17,18]
	23	Guaianin N (Glycoside St-C1)	C_41_H_66_O_12_	Leaf	[16,17,18]
	24	Matesaponin J_2_	C_41_H_66_O_12_	Leaf	[16,17,18]
	25	Echinocystic acid 3-*O*-β-d-glucopyranosyl-(1→3) -*O*-α-*L*-arabinopyranoside	C_41_H_66_O_13_	Leaf	[16,17,18]
	26	Hemslonin A	C_42_H_68_O_13_	Leaf	[16,17,18]
	27	Cussonoside B	C_48_H_78_O_17_	Leaf	[16,17,18]
	28	Oleanolic acid 3-*O*-[β-d-glucopyranosyl-(1→3)]-β-d-galactopyranosyl -(1→2)-*O*-α-*L*-arabinopyranoside (Glycoside St-E2)	C_47_H_76_O_17_	Leaf	[16,17,18]
	29	Ciwujianoside C3	C_53_H_86_O_21_	Leaf	[16,17,18]
	30	Ursolic acid 3-*O*-α-*L*-arabinopyranosyl-28-*O*-α-L-rhamnopyranosyl-(1→4)-*O*-β-d -glucopyranosyl-(1→6)-*O*-β-d-glucopyranoside	C_53_H_86_O_21_	Leaf	[16,17,18]
	31	Oleanolic acid 3-*O*-β-d-glucuronopyranoside	C_36_H_56_O_9_	Leaf, fruit	[11,16,17,18]
	32	Araliasaponin II	C_53_H_86_O_22_	Leaf	[16,17,18]
	33	Begoniifolide A	C_59_H_96_O_26_	Leaf	[16,17,18]
Phenylpropanoids	34	Rosin	C_15_H_20_O_6_	Fruit	[12]
	35	Ferulic acid	C_10_H_10_O_4_	Root	[19]
	36	Caffeic acid	C_9_H_8_O_4_	Root, stem	[19,20]
	37	Syringin	C_17_H_24_O_9_	Root	[19]
	38	*Trans*-coniferin	C_16_H_22_O_8_	Root	[19]
	39	*Trans*-*p*-hydroxycinnamic acid	C_9_H_8_O_3_	Stem	[20]
	40	(*E*)-caffeic acid methyl ester	C_10_H_10_O_4_	Stem	[20]
	41	*Trans*-coniferyl aldehyde	C_10_H_10_O_3_	Stem	[20]
	42	*Trans*-sinapaldehyde	C_11_H_12_O_4_	Stem	[20]
	43	Eugenol glucoside	C_16_H_22_O_7_	Flower	[21]
Caffeoyl quinic acids	44	1,3-di-*O*-caffeoyl quinic acid	C_25_H_24_O_12_	Fruit, root, stem, flower	[13,19,20,21]
	45	1,4-di-*O*-caffeoyl quinic acid	C_25_H_24_O_12_	Fruit, root, stem, flower	[13,19,20,21]
	46	1,5-di-*O*-caffeoyl quinic acid	C_25_H_24_O_12_	Fruit, root, stem, flower, leaf	[13,19,20,21,22]
	47	3,4-di-*O*-caffeoyl quinic acid	C_25_H_24_O_12_	Fruit, flower, leaf	[13,21,22]
	48	3,5-di-*O*-caffeoyl quinic acid	C_25_H_24_O_12_	Fruit, flower, leaf	[13,21,22]
	49	4,5-di-*O*-caffeoyl quinic acid	C_25_H_24_O_12_	Fruit, flower, leaf	[13,21,22]
	50	Methyl chlorogenate	C_17_H_20_O_9_	Fruit	[13]
	51	3-*O*-caffeoyl quinic acid	C_16_H_18_O_9_	Root, stem	[19,20]
	52	4-*O*-caffeoyl quinic acid	C_16_H_18_O_9_	Leaf	[22]
	53	5-*O*-caffeoyl quinic acid	C_16_H_18_O_9_	Root, stem, leaf	[19,20,22]
	54	3,5-dicaffeoylquinic acid methyl ester	C_26_H_26_O_12_	Flower	[21]
	55	3,4-dicaffeoylquinic acid methyl ester	C_26_H_26_O_12_	Flower	[21]
	56	1,3-dicaffeoylquinic acid methyl ester	C_26_H_26_O_12_	Flower	[21]
Flavonoids	57	Quercetin-3-*O*-β-d-glucopyranoside	C_21_H_20_O_12_	Fruit, flower, leaf	[13,21,22,23]
	58	Quercetin-3-*O*-β-d-galactopyranoside	C_21_H_20_O_12_	Fruit	[13]
	59	Rutin	C_27_H_30_O_16_	Fruit, flower, leaf	[13,21,22,23]
	60	Kaempferol-3-*O*-β-d-glucoside	C_21_H_20_O_11_	Fruit, flower	[12,21]
	61	Kaempferol-3-rutinoside	C_27_H_30_O_15_	Fruit, flower, leaf	[12,21,22]
	62	Kaempferol-3-*O*-α-*L*-rhamnoside	C_21_H_20_O_10_	Flower	[21]
	63	Kaempferol	C_15_H_10_O_6_	Flower, leaf	[21,23]
	64	Quercetin	C_15_H_10_O_7_	Leaf	[22,23]
	65	Quercetin-3,7-di-β-*O*-glucopyranoside	C_28_H_34_O_17_	Leaf	[22]
Lignans	66	(−)-Pinoresinol 4-*O*-β-d-glucopyranoside	C_26_H_32_O_11_	Fruit	[12]
	67	(+)-Simplexoside	C_26_H_30_O_11_	Fruit	[12]
	68	(−)-Sesamin	C_20_H_18_O_6_	Fruit, root, stem	[12,19,20]
	69	(−)-Kobusin	C_21_H_22_O_6_	Fruit	[11]
	70	Styraxlignolide E	C_26_H_32_O_11_	Fruit	[12]
	71	Styraxlignolide D	C_26_H_32_O_11_	Fruit	[12]
	72	Helioxanthin	C_20_H_12_O_6_	Root	[19]
	73	Savinin	C_20_H_16_O_6_	Root	[19]
	74	Taiwanin C	C_20_H_12_O_6_	Root	[19]
	75	(+)-*threo*-(7*R*,8*R*)-guaiacylglycerol-β-coniferyl aldehyde ether	C_20_H_22_O_7_	Root	[19]
	76	(+)-*erythro*-(7*S*,8*R*)-guaiacylglycerol-β-coniferyl aldehyde ether	C_20_H_22_O_7_	Root	[19]
	77	Dihydrosesamin-9-*O*-β-d-glucopyranoside	C_26_H_30_O_11_	Flower	[21]
	78	Syringaresinol diglucoside (Eleutheroside E)	C_34_H_46_O_18_	Root	[15,24,25]
	79	Syringaresinol	C_22_H_26_O_8_	Root	[24]
Steroids	80	Stigmasterol	C_29_H_48_O	Root, stem, leaf	[15,19,20,23]
	81	β-sitosterol	C_29_H_50_O	Root, stem	[15,19,20,24]
	82	Daucosterol	C_35_H_60_O_6_	Root, leaf	[14,23]
	83	Stigmasterol-3-*O*-β-d-glucopyranoside	C_35_H_58_O_6_	Leaf	[26]
Fatty acids	84	Behenic acid	C_22_H_44_O_2_	Root	[19]
	85	Undecanedioic acid, monomethyl ester	C_14_H_26_O_4_	Stem	[20]
	86	Octacosanic acid	C_28_H_56_O_2_	Root	[24]
	87	Fumaric acid	C_4_H_4_O_4_	Leaf	[16]
	88	Melissic acid	C_30_H_60_O_2_	Leaf	[23]
	89	Lacceroic acid	C_32_H_64_O_2_	Leaf	[23]
	90	Palmitic acid	C_16_H_32_O_2_	Leaf	[23]
	91	Gheddic acid	C_34_H_68_O_2_	Leaf	[23]
Other compounds	92	5-hydroxymethyl-2-furaldehyde	C_6_H_6_O_3_	Fruit	[12]
	93	5-hydroxymaltol	C_6_H_6_O_4_	Fruit	[12]
	94	Protocatechuic acid	C_7_H_6_O_4_	Fruit	[12]
	95	6-methoxy-7-hydroxycoumarin	C_10_H_8_O_4_	Fruit, root, stem	[12,19,20]
	96	Phenylmethyl-β-d-glucopyranoside-6′-*O*-acetate	C_15_H_20_O_7_	Fruit	[12]
	97	Adenosine	C_10_H_13_N_5_O_4_	Root	[19]
	98	*p*-hydroxybenzoic acid	C_7_H_6_O_3_	Stem	[20]
	99	Syringaldehyde	C_9_H_10_O_4_	Stem	[20]
	100	Vanillin	C_8_H_8_O_3_	Stem	[20]
	101	1-*O*-β-d-glucopyranosyl-(2*S*,3*S*,4*R*,8*E*/*Z*)-2-(2′-hydrooxypalmitoyla mino)-8-octadecene-1,3,4-triol	C_40_H_77_NO_10_	Leaf	[16]
	102	Glyceroyl-1,6,8-trihydroxy-3-methyl-9,10-dioxo-2-anthracene carboxylate	C_19_H_16_O_9_	Leaf	[16]

**Table 2:** Biological activities of the *Acanthopanax henryi* shown by in vitro studies.

Biological Activity	In Vitro Studies		Ref.
	Cell/Bacteria Model	Effects	
Anti-neuroinflammatory	LPS-stimulated BV2 microglia	↓ NO, PGE_2_, IL-1β, TNF-α production; ↓ iNOS, COX-2 expression, ↓ p38 MAPK phosphorylation	[11,12,19,20,21]
Anti-adipogenic	3T3-L1 cells	↑ AMPK-↓ PPARγ-↓ C/EBPα mechanism	[30]
Anti-inflammatory	LPS-stimulated RAW264.7 macrophages	↓ NO, PGE_2_, IL-6, IL-1β, TNF-α production; ↓ iNOS, COX-2 expression, ↓ TLR4-NF-κB, MAPKs phosphorylation; ↓ NF-κB/p65 translocation	[11,20,21,31,32,33]
Antimicrobial	MRSA	MIC, time-kill growth curves, OD600, damage to the cell wall, broken cell membranes and cell lysis, ↓ PBP2a expression	[34,35]
Anticancer	HL-60, HT-29, A549 cells	↓ Cell viability	[14,36]
Anti-oxidant	-	↑ DPPH, O2(−), ABTS scavenging activity	[22,36,37,38]
Anti-AChE	-	↑ AChE inhibitory activity	[22,36,37]
Anti-BuChE	-	↑ BuChE inhibitory activity	[37]
Anti-hyaluronidase	-	↑ hyaluronidase inhibitory activity	[36,38]

Legend: lipopolysaccharide (LPS); nitric oxide (NO); prostaglandin E_2_ (PGE_2_); inducible nitric oxide synthase (iNOS); cyclooxygenase-2 (COX-2); interleukin (IL)-1β; tumor-necrosis factor (TNF)-α; mitogen-activated protein kinase (MAPK); toll-like receptor 4 (TLR4); nuclear factor (NF)-κB; peroxisome proliferator-activated receptor γ (PPARγ); CCAAT/enhancer-binding protein α (C/EBPα); AMP-activated protein kinase (AMPK); minimum inhibitory concentration (MIC); optical density at 600 nm (OD600); penicillin-binding protein 2a (PBP2a); methicillin-resistant *Staphylococcus aureus* (MRSA); 2,2-diphenyl-1-picrylhydrazyl radical (DPPH); 2,2-azino-bis-3-ethyl benzthiazoline-6-sulphonic acid (ABTS); acetyl cholinesterase (AChE); butyryl cholinesterase (BuChE); ↑ increase, ↓ decrease.
